# TFIIIB Subunit Bdp1 Participates in RNA Polymerase III Transcription in the Protozoan Parasite* Leishmania major*

**DOI:** 10.1155/2019/1425281

**Published:** 2019-04-01

**Authors:** Fiordaliso C. Román-Carraro, Luis E. Florencio-Martínez, Gabriela Romero-Meza, Tomás Nepomuceno-Mejía, Julio C. Carrero, Rossana Arroyo, Jaime Ortega-López, Rebeca G. Manning-Cela, Santiago Martínez-Calvillo

**Affiliations:** ^1^Unidad de Biomedicina, Facultad de Estudios Superiores Iztacala, Universidad Nacional Autónoma de México, Av. de Los Barrios 1, Col. Los Reyes Iztacala, Tlalnepantla, Estado de México, CP 54090, Mexico; ^2^Departamento de Inmunología, Instituto de Investigaciones Biomédicas, Universidad Nacional Autónoma de México, Ciudad de México, CP 04510, Mexico; ^3^Departamento de Infectómica y Patogénesis Molecular, Centro de Investigación y de Estudios Avanzados del IPN, Av. IPN 2508, Ciudad de México, CP 07360, Mexico; ^4^Departamento de Biotecnología y Bioingeniería, Centro de Investigación y de Estudios Avanzados del IPN, Av. IPN 2508, Ciudad de México, CP 07360, Mexico; ^5^Departamento de Biomedicina Molecular, Centro de Investigación y de Estudios Avanzados del IPN, Av. IPN 2508, Ciudad de México, CP 07360, Mexico

## Abstract

*Leishmania major,* a protozoan parasite that diverged early from the main eukaryotic lineage, exhibits unusual mechanisms of gene expression. Little is known in this organism about the transcription factors involved in the synthesis of tRNA, 5S rRNA, and snRNAs, transcribed by RNA Polymerase III (Pol III). Here we identify and characterize the TFIIIB subunit Bdp1 in* L. major* (LmBdp1). Bdp1 plays key roles in Pol III transcription initiation in other organisms, as it participates in Pol III recruitment and promoter opening.* In silico *analysis showed that LmBdp1 contains the typical extended SANT domain as well as other Bdp1 conserved regions. Nevertheless, LmBdp1 also displays distinctive features, including the presence of only one aromatic residue in the N-linker region. We were not able to produce null mutants of LmBdp1 by homologous recombination, as the obtained double replacement cell line contained an extra copy of LmBdp1, indicating that LmBdp1 is essential for the viability of* L. major* promastigotes. Notably, the mutant cell line showed reduced levels of the LmBdp1 protein, and its growth was significantly decreased in relation to wild-type cells. Nuclear run-on assays demonstrated that Pol III transcription was affected in the mutant cell line, and ChIP experiments showed that LmBdp1 binds to 5S rRNA, tRNA, and snRNA genes. Thus, our results indicate that LmBdp1 is an essential protein required for Pol III transcription in* L. major*.

## 1. Introduction

In eukaryotic cells, transcription of nuclear DNA is carried out by three different classes of RNA polymerases (Pol): Pol I, II, and III. Each of these enzymes transcribes a specific group of genes, and each depends on transcription factors to bind its promoter regions. Pol I produces 18S, 5.8S, and 28S rRNAs [1], while Pol II generates mRNAs and most microRNAs and snRNAs [2]. Pol III transcribes genes encoding 5S rRNA, tRNAs, U6 snRNA, 7SL RNA, and other small essential RNA molecules [3, 4]. Pol III promoters have been divided into three main types: 5S rRNA genes possess a type 1 promoter, composed of three gene-internal elements (boxes A and C and intermediate element); type 2 promoters are present on tRNA genes, and they consist of two gene-internal sequences (boxes A and B); and U6 snRNA genes contain type 3 promoters, which are constituted by three small sequence elements located upstream of the genes (TATA box, proximal sequence element, and distal sequence element) [5].

General transcription factors TFIIIA, TFIIIB, and TFIIIC are required for accurate initiation of Pol III transcription [6]. TFIIIB is needed to form a transcriptionally active preinitiation complex (PIC) at all three types of promoter regions, as it participates in Pol III recruitment and promoter opening [7]. TFIIIB consists of three subunits: the TATA-binding protein (TBP), the TFIIB-related factor 1 (Brf1), and B double prime 1 (Bdp1) [8]. Notably, Bdp1 is specific to Pol III transcription, since it does not show homology with any of the general transcription factors of other RNA polymerases. Early studies showed that Bdp1 contains a SANT (Swi3, Ada2, N-Cor, and TFIIIB) domain, which is a highly conserved ~50-amino acid motif present in several proteins involved in transcriptional regulation [9]. However, recent analysis established that the SANT domain present in Bdp1 from human and yeast is actually larger and possesses other distinguished features, and accordingly it was renamed extended SANT domain [10–12]. In yeast, the Bdp1 extended SANT domain is essential for cell viability, as it is needed for Pol III PIC assembly and stability [13]. The extended SANT domain interacts directly with DNA and with subunits Brf1 and TBP [14, 15]. Other regions of Bdp1 associate with subunits C128, C37, and C34 of Pol III [11, 13, 16] and subunit Tfc4 of TFIIIC [17, 18]. Thus, Bdp1 establishes an intricate network of interactions within the Pol III PIC.

The trypanosomatid parasite* Leishmania major* is the etiological agent of cutaneous leishmaniasis in Asia and Africa [19]. The parasite is transmitted to humans through the bite of infected sandflies of the genus* Phlebotomus*. In addition to their medical importance,* L. major *and other trypanosomatid protozoa, such as* Trypanosoma brucei* and* Trypanosoma cruzi*, are relevant because they express their genes by uncommon processes, including Pol II polycistronic transcription and trans-splicing [20–22].

Little is known in trypanosomatids about either the DNA sequences or the proteins that participate in Pol III transcription initiation. Unlike other organisms, all snRNA genes are transcribed by Pol III in these parasites [23]. Notably, snRNA expression in* T. brucei* and* L. major* is controlled by extragenic boxes A and B located within tRNA or tRNA-like sequences [24, 25]. Although 5S rRNA genes contain the characteristic gene-internal elements, their promoter activity has not been proven [26]. Neither TFIIIA nor TFIIIC has been identified in trypanosomatids [27]. However, orthologues of TFIIIB subunits TBP and Brf1 have been studied in* T. brucei*. TBP, which participates in transcription by all three RNA polymerases, has been primarily investigated in the context of Pol II transcription of the spliced-leader (SL) RNA genes [28, 29]. On the other hand, knockdown analysis by RNA interference demonstrated that Brf1 is indeed involved in Pol III transcription and that it is indispensable for cell survival of procyclic forms of* T. brucei *[30]. Another protein that participates in the regulation of Pol III transcription in* T. brucei* is Maf1, which inhibits tRNA and snRNA transcription by associating with their promoter regions [31].

Here we identified and characterized the TFIIIB subunit Bdp1 in* L. major* (LmBdp1). Bioinformatic analysis shows that LmBdp1 possesses the characteristic extended SANT domain and other conserved regions that flank this domain. Attempts to generate null mutants produced a cell line with an additional copy of LmBdp1. Nevertheless, Western blot analysis showed that the levels of LmBdp1 were considerably diminished in the mutant cell line. Notably, the growth of this cell line was significantly reduced in relation to wild-type cells. Nuclear run-on assays demonstrated that Pol III transcription was affected in the mutant cell line, and ChIP experiments showed that LmBdp1 binds to 5S rRNA, tRNA, and snRNA genes.

## 2. Materials and Methods

### 2.1. In Silico Analyses

Sequences were obtained from TriTrypDB database (release 40) (http://tritrypdb.org/tritrypdb/), from the National Center for Biotechnology Information (NCBI) database (http://www.ncbi.nlm.nih.gov), and from Universal Protein Resource (UniProt). Multiple sequence alignments were made with the Clustal Omega program (http://www.ebi.ac.uk/Tools/msa/clustalo/) and shaded manually. To predict the protein secondary structure, the PSIPRED Protein Sequence Analysis Workbench (http://bioinf.cs.ucl.ac.uk/psipred/) was used. Homology modeling was performed with SWISS-MODEL v 3.7 (http://swissmodel.expasy.org/interactive), Swiss-PDB Viewer program (http://www.expasy.org/spdbv/), and PyMOL v 2.1.1 (https://pymol.org/2/) using the crystallographic structure of human extended SANT domain (PDB id: 5n9g) and yeast Bdp1 (PDB id: 6F41) as templates. Phosphorylated residues were predicted with PhosTryp (http://phostryp.bio.uniroma2.it/), which was developed for the specific identification of phosphorylated residues in Trypanosomatid proteins, as the method has been trained using phosphoproteomic data from* Leishmania, T. brucei*, and* T. cruzi* [32].

### 2.2. Cell Culture and Transfection

Promastigotes from* L*.* major* MHOM/IL/81/Friedlin (LSB-132.1) were grown in BM medium (1× M199 medium pH 7.2 containing 10% heat-inactivated fetal bovine serum, 9.5 g/l brain heart infusion, 40 mM HEPES, 0.01 mg/ml hemin, 0.0002% biotin, 100 IU/ml penicillin, 100 *μ*g/ml streptomycin, and 1× L-glutamine) at 26°C and harvested in the mid-log phase. To generate the* L. major* cell line that expresses LmBdp1 fused to a PTP tag, wild-type promastigotes were transfected by electroporation with the LmBdp1-PTP vector. Briefly, 4 × 10^7^ promastigotes in 0.4 ml electroporation buffer (25 mM HEPES, 120 mM KCl, 0.15 mM CaCl_2_, 5 mM MgCl_2_, 10 mM KH_2_PO_4_, 10 mM K_2_HPO_4_, 2 mM EDTA, pH 7.6) were transfected with 10 *μ*g of LmBdp1-PTP by electroporation at 1600 V, 50 *μ*F, and 25 Ω (BTX Electro Square Porator ECM 830). Cells were grown for 24 h before spreading on plates containing 0.7% Seaplaque GTG agarose (FMC Bioproducts) in BM medium with 50 *μ*g/ml G418. Some of the isolated colonies were selected for further analysis. The same electroporation conditions were used for transfections with the* pac* and* hyg* targeting cassettes, for the generation of knockout parasites.

### 2.3. Plasmid Constructs

To generate the LmBdp1-PTP vector, for immunofluorescence and ChIP assays, the RPB6 gene from the pB6-PTP plasmid [26] was substituted with the LmBdp1 gene. To that end, pB6-PTP was digested with* Xma*I and* Xba*I and the RBP6 gene was eliminated by agarose gel electrophoresis. The LmBdp1 gene (*LmjF36.6530*) (without the terminal codon) was amplified by PCR using primers Bdp1PTP-for-*Xma*I (5′-ACCCGGGATGGACGACAACGAGTTCGA) and Bdp1PTP-rev-*Xba*I (5′-ATCTAGACTCAAACGAGAAGTCCGAGT). The PCR product was cloned into pGEM-T Easy vector, digested with* Xma*I and* Xba*I, and ligated into the pB6-PTP backbone. To produce plasmid pΔBdp1-pac for knockout experiments, the* pac*-gene insert (1.7 kb) was obtained by digesting pΔ75-pac [33] with* Eco*RI and* Sac*I. The 5′ targeting region from LmBdp1 (566 bp) was amplified by PCR with oligonucleotides Bdp1-for-*Xba*I (5′-ATCTAGAGTCGGCCGTTGCGCTTCTC) and Bdp1-rev-*Eco*RI (5′-AGAATTCGGCTCCACCGGTGCGTATC). The 3′ targeting region (550 bp) was amplified with primers Bdp1-for-*Sac*I (5′-AGAGCTCCGGTAGAACCAAAAAGTTCG) and Bdp1-rev-*Xho*I (5′-ACTCGAGTATAAGTCCGACAGCGAAGA). To produce vector pΔBdp1-hyg, the* hyg*-gene fragment (1.8 kb) from pΔ75-hyg [33] was obtained after* Spe*I and* Sac*I digestion. The 5′ targeting region was amplified with primers Bdp1-for-*Xba*I and Bdp1-rev-*Spe*I (5′-AACTAGTGGCTCCACCGGTGCGTATC); and the 3′ targeting region was amplified with oligonucleotides Bdp1-for-*Sac*I and Bdp1-rev-*Xho*I. The PCR products from 5′ and 3′ targeting regions were digested with the restriction enzymes indicated in the name of each oligonucleotide. For both knockout vectors, the selectable-marker gene and the two flanking regions were cloned into pBluescript II KS (digested with* Xba*I and* Xho*I), by a single four-fragment ligation step. To obtain the targeting cassettes, pΔBdp1-pac and pΔBdp1-hyg were digested with* Xba*I and* Xho*I. For nuclear run-on experiments, fragments of* L. major* genes transcribed by the three different RNA polymerases were amplified by PCR and cloned into the pGEM-T Easy vector. The 24S*β* rRNA gene (*LmjF.27.rRNA.26*; 113 bp) was amplified with primers LmrRNA24S*β*-5′ (5′-TTCCGGAGTCTTGTTTCGAG) and LmrRNA24S*β*-3′ (5′-GTGGATTCGGTTGGTGAGTTG); and the 18S rRNA gene (*LmjF.27.rRNA.01*; 370 bp) was amplified with oligonucleotides Lm-rRNA18S-5′ (5′-CGGCCTCTAGGAATGAAGG) and LmrRNA18S-3′ (5′-CCCCTGAGACTGTAACCTC). The *α*-tubulin gene (*LmjF.13.0330*; 338 bp) was amplified with primers alfa-tub-5′ (5′-AGAAGTCCAAGCTCGGCTACAC) and alfa-tub-3′ (5′-GTAGTTGATGCCGCACTTGAAG); and the spliced-leader RNA gene (*LmjF.02.SLRNA.0010*; 303 bp) was amplified with primers SL-5′ (5′ GAGCGCGGTGGGCATGACA) and SL-3′ (5′-ACTGCAAGGGTGCGCCCG). The* LmjF.06.0370* gene (521 bp) was amplified with oligonucleotides Lm06-0370-5′ (5′-GAAGCGATGGACTGTTCTGG) and Lm06-0370-3′ (5′-CGGTCCTTGCTGCGAATATC); and the* LmjF.06.0210* gene (503 bp) was amplified with primers Lm06-0210-5′ (5-GCCGGAGACATTTGCGTAC) and Lm06-0210-3′ (5′-CTATGGCGACGGGATCATC). The* LmjF.06.0200* gene (547 bp) was amplified with primers Lm06-0200-5′ (5′-CCATCCCATGACAAGAGC) and Lm06-0200-3′ (5′-TGTAGTCGCTGTACTCGC); and the tRNA-Phe gene (*LmjF.09.TRNAPHE.01*; 338 bp) was amplified with oligonucleotides Lm09-TRNAPHE-5′ (5′-TTCATCCGCGCAAAGAGG) and Lm09-TRNAPHE-3′ (5′-GGCCTTCCACGTATTTCG). The tRNA-Tyr gene (*LmjF.36.TRNATYR.01*; 316 bp) was amplified with primers Lm36-TRNATYR-5′ (5′-AGTGCCGAGAAGTTCGACG) and Lm36-TRNATYR-3′ (5′-TCGTCTCCGTTCCTGTTGC); and the 5S rRNA gene (*LmjF.15.5SrRNA.01*; 344 bp) was amplified with oligonucleotides Lm15-rRNA5S-5′ (5′-GAAAGCATCTCTGTG GGTTCGA) and Lm15-rRNA5S-3′ (5′CCCGGGGTCCTGCAAATG). The U2 snRNA gene (*LmjF.31.snRNA.01*; 127 bp) was amplified with primers U2-5′ (5′-AAACGTGGAACTCCAAGGAA) and U2-3′ (5′-TATCTTCTCGGCTATTTAGC); and the tRNA-Sec gene (524 bp) was amplified with primers Lm-TRNASEC524-5′ (5′-CCGGCTGCCTTCATCAACTC) and Lm-TRNASEC524-3′ (5′-GCGCATACGTTTCGGAGTCC). After transformation of JM109 competent cells, plasmid DNA was purified with NucleoSpin plasmid columns (Macherey-Nagel) as specified by the supplier. The identity of each insert was confirmed by sequencing using the T7 and SP6 primers.

### 2.4. Indirect Immunofluorescence Assays

The cellular localization of LmBdp1 labeled with a PTP tag was determined by indirect immunofluorescence, as previously described [26, 34]. For these assays, 1.5 × 10^7^ mid-log promastigotes were incubated with a rabbit anti-Prot C polyclonal antibody (Delta Biolabs) and a secondary goat anti-rabbit antibody conjugated with Alexa-Fluor 488 (Life Technologies). Images were obtained with a Zeiss Axio Vert.A1 epifluorescence microscope and analyzed with the ZEN 2012 software (Blue edition).

### 2.5. Southern Blot and PCR Analyses

For Southern blot experiments, 5 *μ*g of genomic DNA was digested with* Xho*I and* Sac*I (for analysis of single-knockout parasites), or* Pst*1 (for examination of double-knockout cells). The DNA was separated by electrophoresis on 0.8% agarose gels and transferred to Hybond-NC membranes (GE Healthcare) by capillary action. Blots were hybridized with the 5′ targeting region from LmBdp1 (566 bp) labeled with [*α*-^32^P]dCTP using the High Prime labeling system (Roche). Hybridizations were performed in 50% formamide, 5× SSC, 0.2% SDS, and 4× Denhardt's reagent at 42°C. Filters were washed at 68°C in 0.2× SSC and 0.1% SDS. To verify the replacement of the LmBdp1 gene with the* pac *gene, PCR analysis was carried out with oligonucleotides PAC-LOC3′-REV (5′-GTGGGCTTGTACTCGGTCATGG) and Bdp1-for-upstream (5′-TGTTGGCAACTTGCCACCGT) which recognizes sequences located upstream of the 5′ targeting region; and primers PAC-DHFR-3′-REV (5′-GGAGGGAGGAATGAGGTGAGCT) and Bdp1-for-upstream. The correct integration of the* hyg *gene was confirmed by PCR with primers HYG-rev (5′-GTCGGAGACGCTGTCGAACT) and Bdp1-for-upstream.

### 2.6. Generation of LmBdp1 Polyclonal Antibody

Competent cells of* Escherichia coli* BL21 (DE3) were transformed with plasmid pCold-LmBdp1. Expression of LmBdp1 recombinant protein (LmBdp1r) was induced with 1 mM isopropyl *β*-D-1-thiogalactopyranoside (IPTG) at 37°C for 18 h. Affinity chromatography with Ni-Sepharose 6 Fast Flow matrix (GE Healthcare) was carried out to purify the LmBdp1r protein, according to the manufacturer's specifications. Around 50 *μ*g/animal of purified LmBdp1r was employed to inoculate subcutaneously six-week-old male BALB-C mice in TiterMax Gold adjuvant (Sigma) at a 1:1 ratio. Pre-immune normal mouse serum was collected before inoculation. Serum was collected six weeks after antigen immunization. Western blot analyses against LmBdp1r and protein extracts from promastigotes were performed to confirm the specificity of the anti-LmBdp1 polyclonal antibody.

### 2.7. Western Blot Analysis

Whole-cell protein extracts were obtained by standard protocols [35]. Briefly, 2 ×10^8^ parasites were lysed in RIPA buffer (150 mM NaCl, 0.5% sodium deoxycholate, 0.1% SDS, 50 mM Tris pH 7.4, and 1× protease inhibitors). The extract was incubated for 30 min on ice and mixed every ten minutes and then centrifuged at 1500 × g for 10 min. The supernatant was recovered and stored at −70°C. For Western blot analysis, 50 *μ*g of total protein was fractionated by 10% SDS-PAGE and blotted onto PVDF membranes (Bio-Rad). Membranes were incubated with rabbit Peroxidase Anti-Peroxidase (PAP) (1:1600 dilution, Sigma), polyclonal anti-LmBdp1 antiserum (1:100 dilution), or polyclonal human *β*-tubulin antibody (1:500, Invitrogen). Proteins were detected with a horseradish peroxidase-conjugated secondary antibody and developed using an ECL kit (GE Healthcare).

### 2.8. Nuclear Run-On Experiments

These assays were performed as described before [30, 36] with isolated nuclei from 2 × 10^8^ mid-log promastigotes. Labeled nascent RNA was hybridized to Hybond-N+ membranes (GE Healthcare) containing dots of 2 *μ*g of plasmid DNA. These plasmids possess fragments of genes transcribed by Pol I (18S and 24S*β* rRNA), Pol II (*α*-tubulin, spliced-leader RNA,* LmjF.06.0200*,* LmjF.06.0210*, and* LmjF.06.0300*), Pol III (5S rRNA, U2 snRNA, tRNA-Phe, and tRNA-Tyr), and by both Pol II and III (tRNA-Sec). Hybridization was performed for 48 h at 50°C in 50% formamide, 5× SSC, 0.2% SDS, 4× Denhardt's reagent, and 100 *μ*g/ml salmon sperm DNA. Filters were washed to a final stringency of 0.1× SSC and 0.1% SDS at 65°C. RNA hybridization signals were quantified by densitometry using the MultiGauge software.

### 2.9. Chromatin Immunoprecipitation Assays

The ChIP procedures were performed as previously described [31]. Briefly, 2 × 10^8^ promastigotes were cross-linked with formaldehyde (final concentration of 1%) for 5 min at 37°C. A Vibra-Cell VCX130 ultrasonic processor (Sonics) was employed to lyse the cells (15 s on/off, 40% amplitude, for 5 min). Nuclei were pelleted and resuspended in sonication buffer (1% SDS, 10 mM EDTA, 50 mM Tris-HCl pH 8.1). Chromatin was sonicated to an average DNA size of about 200 to 500 bp with a BioRuptor UCD-200 (Diagenode) (30 s on/30 s off, high intensity) for 30 cycles. The sonicated material was pre-cleared by adding protein A/G plus-agarose beads (Santa Cruz Biotechnology) and mixing for 1 h at 4°C. Chromatin samples were incubated overnight at 4°C with rabbit anti-Prot A antibody (Sigma) or nonspecific rabbit immune serum (negative control). Protein-DNA complexes were incubated for 1 h with protein A/G plus-agarose beads and 200 ng of sonicated salmon sperm DNA and washed as previously described [37]. Cross-links were reversed with 200 mM NaCl at 65°C overnight. Samples were treated with RNase A and proteinase K. DNA was precipitated with sodium acetate and ethanol and quantified. Each ChIP experiment was performed at least three times.

### 2.10. Quantitative Real-Time PCR Experiments

The Platinum SYBR Green qPCR Super Mix-UDG kit (Invitrogen) was employed to examine around 5 ng of immunoprecipitated DNA by quantitative real-time PCR. The results were analyzed by the 2^−ΔΔCq^ method, as reported previously [30, 37]. Reactions were carried out with optimized conditions that produce a single amplicon of the correct size, in triplicate. Results are presented as fold enrichment over negative control precipitations. The promoter region of the 18S rRNA gene (*LmjF.27.rRNA.01*) was amplified with primers 18Sp-for (5′-TTGTTTGGGTGGAGGTGAGA) and 18Sp-rev (5′-CAAAATCATCAAACCCCGTTC). The promoter of the spliced-leader RNA gene (*LmjF.02.SLRNA.0010*) was amplified with oligonucleotides LmjF-SL-PromF (5′-GAGCGCGGTGGGCATGACA) and LmjF-SL-PromR (5′-AAGCCATCACCACCGCAGC). The *α*-tubulin gene (*LmjF13.0330*) was amplified with primers TUB-for (5′-AGAAGTCCAAGCTCGGCTACAC) and TUB-rev (5′-GTAGTTGATGCCGCACTTGAAG). The 5S rRNA gene (*LmjF.11.5SRRNA.03*) was amplified with primers 5S-for (5′-GAAAGCATCTCTGTGGGTTCGA) and 5S-rev (5′-AACCCTGAGTGCCGTACTC). The U2 snRNA gene (*LmjF.31.snRNA.01*) was amplified with oligonucleotides U2-for (5′-TATCTTCTCGGCTATTTAGC) and U2-rev (5′-AAACGTGGAACTCCAAGGAA). The tRNA-Ala gene (*LmjF.31.TRNAALA.01*) was amplified with primers Ala-for (5′-ATTGGGACGTTACCGCGTCG) and Ala-rev (5′-ATTGCGGCCCAGGCCTTTCA). The tRNA-like associated to the U2 snRNA gene was amplified with primers Like-for (5′-CCGAGAAGATATGTTAGTACCACC) and Like-rev (5′-AGGAAAAGATGCTTTCGACGAG). The tRNA-Met gene (*LmjF.11.TRNAMET.01*) was amplified with oligonucleotides tRNAmet-F (5′-AAAGTTTGCGACCGGTGAG) and tRNAmet-R (5′-CACAACTTTCACTCGTAGCCG). The U4 snRNA (*LmjF.36.snRNA.01*) was amplified with primers U4-5′ (5′-AAGCCTTGCGCAGGGAGATGT) and 5′U4Lmjend (5′-GACAAAAAGTAGTCCCCACCC). The tRNA-like associated to the U4 snRNA was amplified with oligonucleotides U4tRNA-like 5′ (5′-GAAAAAAGGAGCGCCGCCCCA) and U4tRNA-like 3′ (5′-CGCAAGGCTTGCCTTGGGTGT).

## 3. Results

### 3.1. LmBdp1 Possesses the Extended SANT Domain and Other Conserved Regions

**Figure 1:**
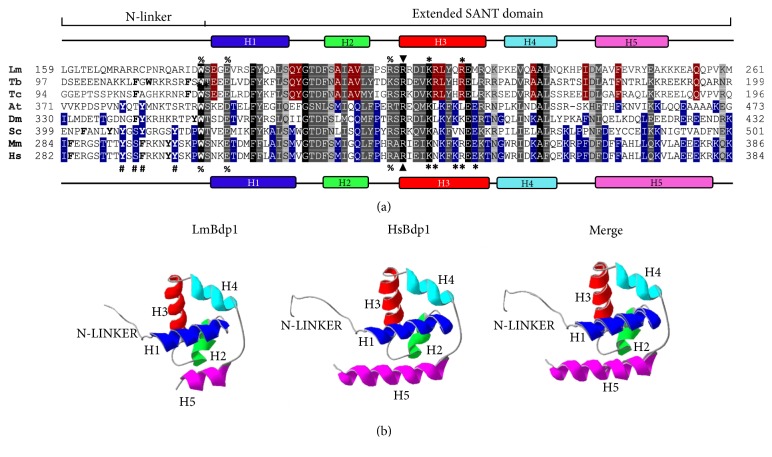
Sequence and structure analyses of the extended SANT and N-linker domains from LmBdp1. (a) Multiple sequence alignment of the N-linker and extended SANT domains from* L. major* (Lm),* T. brucei* (Tb),* T. cruzi* (Tc),* A. thaliana* (At),* D. melanogaster *(Dm),* S. cerevisiae* (Sc),* M. musculus* (Mm), and* H. sapiens* (Hs). Identical residues in all species are indicated by black shading. Conserved substitutions are denoted by dark-grey shading with white lettering and semiconserved substitutions are indicated by light-grey shading with black lettering, according to the Clustal Ω program. Trypanosomatid-specific conserved residues are shaded in red with white letters. Conserved amino acids in at least three species different from trypanosomatids (At, Dm, Sc, Mm, and Hs) are shaded in blue with white lettering. In the N-linker, aromatic residues are denoted in bold. Below the Hs sequence, the hash characters (#) indicate conserved residues that interact with the DNA minor groove (Y291, S293, F294, and Y299 in human Bdp1). The percent sign (%) indicates highly conserved amino acids (W303, E307, and R332 in human Bdp1) involved in the interaction between the N-linker and the extended SANT domain. The triangle symbol (▲) shows the invariably conserved R334 that forms a salt bridge with residue E191 from TBP and also interacts with the template DNA strand. Asterisks (*∗*) indicate conserved amino acids K338, N339, K342, R343, and E345 that contact both faces of the major groove of the DNA. Symbols above the Lm sequence indicate conserved residues in LmBdp1. Predicted *α*-helices are denoted by rectangles (H1 to H5) for LmBdp1 (above the alignment) and human Bdp1 (below the alignment). (b) Predicted three-dimensional structure of the N-linker and extended SANT domain of LmBdp1 by homology-modeling using the crystal structure of human Bdp1 (HsBdp1) as a template. The colors of the five *α*-helices are conserved in panels (a) and (b).

Based on the presence of the SANT domain, gene* LmjF36.6530* was identified as a potential orthologue of Bdp1 in* L. major *(LmBdp1) [20]. However, as SANT domains are also present in other proteins, including the subunits of many chromatin-remodeling complexes [9], we further analyzed the sequence and structure of LmBdp1 to verify its identity. Sequence alignments with Bdp1 orthologues from several species showed that LmBdp1 indeed contains the typical extended SANT domain, characterized by the presence of five *α*-helices (Figure 1(a)). Moreover, two sequences that flank the extended SANT domain, the N-linker and the long arm [11, 12], are conserved or semi-conserved in LmBdp1. The N-linker, involved in the binding to the minor groove of the DNA, is distinguished by the occurrence of aromatic residues, including the invariably conserved tryptophan (W) residue that is present in LmBdp1 (Figure 1(a)). Nevertheless, LmBdp1 does not contain other conserved aromatic residues (Y291, F294, and Y299) that are important for the interaction Bdp1-DNA (Figure 1(a)) [10]. The long arm [11], also known as helix C [16] or Bdp1 stem [12], which participates in interactions with Brf1 and subunit C34 of Pol III, is also present in LmBdp1 (Supplementary Figure 1(a)). The tether region, located N-terminally to the N-linker (Supplementary Figure 1), interacts with Pol III subunits C128, C37, and C34 and in most Bdp1 orthologues contains several *β*-sheet structures [11]. However, unlike most species, the tether region in LmBdp1 is not predicted to fold into *β*-sheet structures (Supplementary Figure 1). Other amino acids that are important for the function of human Bdp1 are conserved in LmBdp1. These include R334 (R211 in LmBdp1), K338 (K215 in LmBdp1) and R343 (R220 in LmBdp1) (Figure 1(a)). R334 interacts with TBP, while K338 and R343 contact the major groove of the DNA [10]. The extended SANT domain is also conserved in Bdp1 orthologues in* T. brucei* and* T. cruzi* (Figure 1(a)), as well as in other species of* Leishmania* (Supplementary Figure 2).

To further study the structure of the extended SANT domain present in LmBdp1, its predicted three-dimensional structure was generated by homology modeling, using as templates the crystal structures of Bdp1 from human (Figure 1(b)) and yeast (Supplementary Figure 1(b)). The architectures of the obtained three-dimensional structures for the extended SANT domain of LmBdp1 are very similar to the ones reported for human and yeast [10–12], showing a conserved distribution of the five *α*-helices and the long arm. Thus, these results and the functional data showed below demonstrate that* LmjF36.6530* is indeed the orthologue of Bdp1 in* L. major*.

### 3.2. LmBdp1 Is a Nuclear Protein

**Figure 2:**
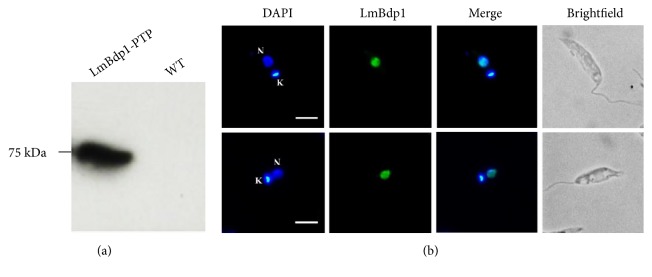
Nuclear localization of LmBdp1. (a) Western blot analysis with proteins isolated from the* L. major* cell line that expresses the LmBdp1-PTP recombinant protein, using an anti-Prot C monoclonal antibody. Wild-type parasites were also analyzed as control. (b) The location of LmBdp1 labeled with a PTP tag was determined by indirect immunofluorescence assays using anti-Prot C monoclonal antibody and an Alexa-Fluor 488 conjugated secondary antibody. Nucleus (N) and kinetoplast (K) are stained with DAPI. Size bars represent 5 *μ*m.

In order to determine the cellular distribution of LmBdp1 in* L. major*, we performed indirect immunofluorescence experiments. To that end, a cell line where LmBdp1 was labeled with a C-terminal PTP tag was generated. The PTP tag is constituted by Protein A (Prot A) and Protein C (Prot C) epitopes separated by a tobacco etch virus (TEV) protease cleavage site [38]. The expression of the recombinant LmBdp1-PTP protein was confirmed by Western blot analysis with an anti-Prot C antibody (Figure 2(a)). Immunofluorescence experiments were carried out on fixed and permeabilized promastigotes with the same antibody. The images obtained demonstrated that LmBdp1 localizes to the nucleus, as anticipated for a protein that regulates transcription (Figure 2(b)). The observed dotted pattern is similar to that obtained with Brf1 in* T. brucei* [30].

### 3.3. Targeted Gene Replacement of LmBdp1

To analyze the effects of the elimination of LmBdp1 on cell growth and transcription in promastigotes of* L. major*, we intended to generate LmBdp1 null mutant parasites by targeted gene replacement. Although* L. major* Friedlin is a diploid organism, some chromosomes are aneuploid [39]. However, it has been shown that this strain possesses two copies of chromosome 36 [40], where the LmBdp1 gene is located. Consequently, two sequential rounds of targeted gene disruption were planned to obtain null mutants of LmBdp1, using plasmids that contain* puromycin* (*pac*) and* hygromycin* (*hyg*) resistance genes. In these plasmids, the selectable marker genes are bounded by 5′ and 3′ flanking regions of LmBdp1.

**Figure 3:**
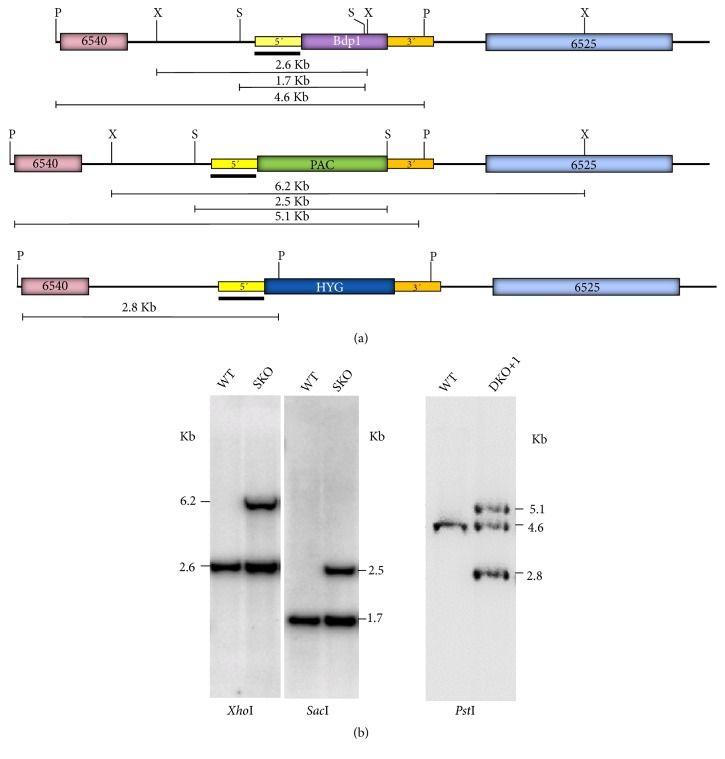
Southern blot analysis of LmBdp1 knockout parasites. (a) Restriction maps of the wild-type LmBdp1 locus (top map) and mutant loci with a copy of LmBdp1 substituted with the* pac* gene (middle map) or replaced with the* hyg* gene (bottom map). Restriction sites for* Xho*I,* Sac*I, and* Pst*I are indicated. Sizes of predicted restriction fragments are shown. The location of the fragment employed as a probe (5′ targeting region) in the Southern blot experiments is denoted with a black bar. (b) Southern blot analysis with genomic DNA isolated from the LmBdp1 single-knockout (SKO) clone digested with* Xho*I and* Sac*I (left figures); and with genomic DNA isolated from the double-knockout +1 (DKO+1) clone digested with* Pst*I (right figure). The 5′ flanking region was used as a probe.

The single-knockout cell line for LmBdp1 was generated by transfecting wild-type promastigotes with the targeting cassette from pΔLmBdp1-pac, and clones were selected with puromycin. Southern blot analysis using the 5′ flanking region as a probe showed a 6.2 kb band, expected from targeted gene replacement, with genomic DNA from a single-knockout clone digested with* Xho*I, in addition to the 2.6 kb band that corresponds to the undisrupted gene (Figure 3). The expected bands of 2.5 kb (gene replacement) and 1.7 kb (undisrupted gene) were also observed with* Sac*I-digested genomic DNA. Moreover, the* pac *gene was amplified by PCR from the single-knockout clone (Supplementary Figure 3). Thus, these results demonstrate that one copy of LmBdp1 was replaced with the* pac* gene.

To obtain the double-knockout cell line for LmBdp1, the single-knockout clone was transfected with the targeting cassette from vector pΔLmBdp1-hyg, and cells were selected with puromycin and hygromycin. The obtained transfected clones showed growth defects (see below). Southern blots of* Pst*I-digested genomic DNA from the double-knockout cell line showed that the second allelic copy of LmBdp1 was replaced with the* hyg* gene (2.8 kb band) (Figure 3). The expected band of 5.1 kb (*pac* gene) was also observed. However, a band of 4.6 kb that corresponds to the undisrupted LmBdp1 gene was also detected (Figure 3). PCR analysis also showed the presence of coding sequences for* hyg* and* pac* in the cell line (Supplementary Figure 3). These results indicated that an additional copy of LmBdp1 was present in the mutant cell line, which was named double-knockout +1 (DKO+1). Thus, these data strongly suggest that LmBdp1 is an essential gene.

### 3.4. The Amount of the LmBdp1 Protein Was Reduced in the Mutant Parasites

**Figure 4:**
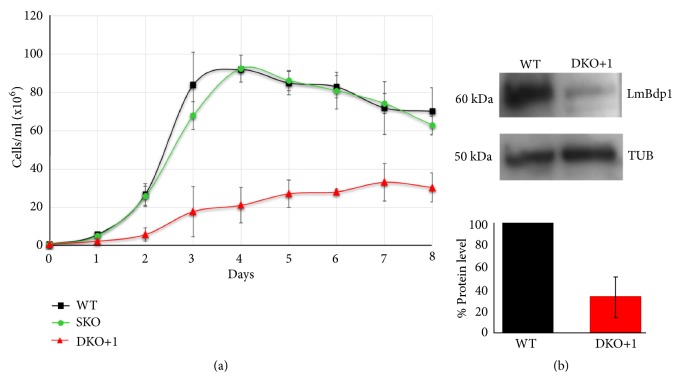
Growth curves and Western blot analysis of LmBdp1 knockout parasites. (a) Growth of promastigotes from wild-type (WT), single-knockout (SKO), and double-knockout +1 (DKO+1) cell lines. Values represent means of three experiments. Standard deviation bars are shown. (b) Western blot analysis of LmBdp1 in wild-type (WT) and double-knockout +1 (DKO+1) parasites using the LmBdp1 antibody. The bands shown here and from two more independent assays were quantified and plotted (lower graph). Protein levels of LmBdp1 were normalized to the amount of *α*-tubulin, which was used as loading control. Standard deviation bars are shown.

To further analyze the growth defect in the mutant cells, growth curves of the DKO+1 cell line were obtained and compared to growth curves of LmBdp1 single-knockout and wild-type parasites (Figure 4(a)). The data showed that the DKO+1 cell line is strongly impaired in growth, as it multiplies more slowly and to a lower stationary-phase cell density than single-knockout and wild-type promastigotes (Figure 4(a)). Growth curves of the single-knockout clone and wild-type parasites did not reveal significant differences (Figure 4(a)).

To determine the level of the LmBdp1 protein in the DKO+1 cell line, recombinant LmBdp1 (LmBdp1r) protein was obtained to produce antibodies against it. To that end, the complete LmBdp1 gene was amplified by PCR and cloned into the pColdI vector, where it was fused to a 6×His tag. The LmBdp1r protein was expressed in* E. coli*, purified by nickel affinity chromatography, and used as antigen for polyclonal antibody production in mice (data not shown). Western blot analysis with the LmBdp1 polyclonal antiserum showed that, comparing to wild-type cells, the amount of LmBdp1 protein was decreased by ~70% in the DKO+1 cell line (Figure 4(b)). Thus, in spite of the presence of an additional copy of the LmBdp1 gene in the DKO+1 parasites, the expression of the LmBdp1 protein is considerably diminished.

### 3.5. Pol III Transcription Is Affected in the Mutant Cell Line

**Figure 5:**
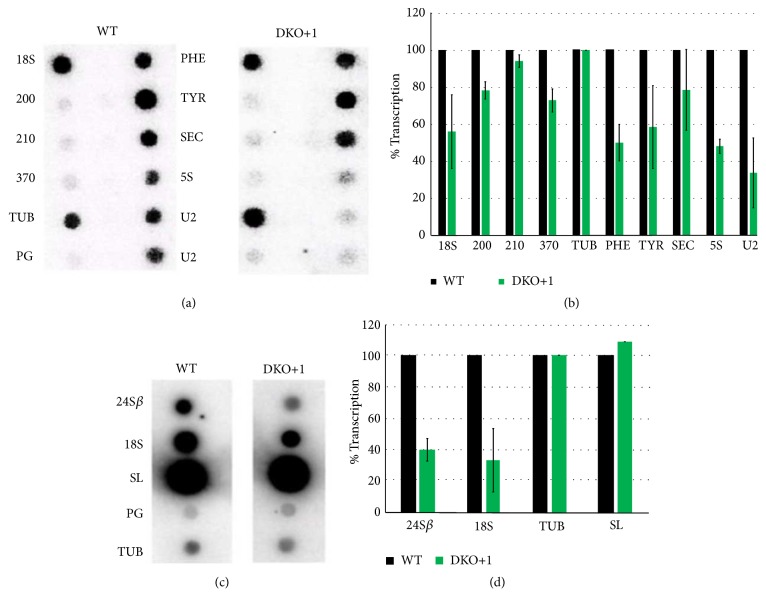
Nuclear run-on analyses with the LmBdp1 double-knockout +1 cell line. (a) Labeled nascent RNA from isolated nuclei from the LmBdp1 double-knockout +1 cell line (DKO+1) and wild-type (WT) parasites was hybridized to dot blots of double-stranded DNAs (2 *μ*g) cloned into pGEM-T Easy. The 18S rRNA gene (18S) was tested as a representative Pol I gene. The Pol II genes analyzed were* LmjF.06.0200 *(200),* LmjF.06.0210 *(210),* LmjF.06.0370* (370), and *α*-tubulin (TUB). The Pol III genes examined were tRNA-Phe (PHE), tRNA-Tyr (TYR), 5S rRNA (5S), and the U2 snRNA (U2), which was examined in duplicate dots. The tRNA-Sec (SEC), transcribed by both Pol II and Pol III, was also analyzed. As control, an empty vector was assessed (PG). (b) The signals obtained for each gene in panel (a) and from two more independent experiments were quantified and plotted, considering as 100% the signal obtained with wild-type promastigotes. Values represent means of the three experiments. Standard deviation bars are shown. RNA levels were normalized to the level of *α*-tubulin. (c) Nuclear run-on analysis performed as indicated in panel (a). The Pol I genes analyzed were 24S*β* rRNA (24S*β*) and the 18S rRNA (18S). The Pol II genes examined were SL RNA (SL) and *α*-tubulin (TUB). As control, an empty vector was also analyzed (PG). (d) The signals obtained for each gene in panel (c) and from two more independent experiments were quantified and plotted, as indicated in panel (b).

To determine if the reduced levels of LmBdp1 have an effect on Pol III transcription, run-on experiments were performed with isolated nuclei from the DKO+1 cell line and wild-type promastigotes (Figure 5). The genes analyzed were tRNA-Phe, tRNA-Tyr, 5S rRNA, and U2 snRNA (transcribed by Pol III); tRNA-Sec (transcribed by Pol II and Pol III); *α*-tubulin,* LmjF.06.0200*,* LmjF.06.0210*, and* LmjF.06.0370* (transcribed by Pol II); and the 18S rRNA (transcribed by Pol I). The hybridization signals observed in Figure 5(a) and two more independent experiments were quantitated, setting to 100% the transcription signal obtained with wild-type cells (Figure 5(b)). Normalization was performed with *α*-tubulin. As anticipated, Pol III transcription was decreased in the DKO+1 cell line, since signal from U2 snRNA, 5S rRNA, tRNA-Phe, and tRNA-Tyr was reduced to 33, 47, 49, and 58% of the control value, respectively (Figures 5(a) and 5(b)). Thus, the nuclear run-on data corroborate the involvement of LmBdp1 in Pol III transcription. Signal of the tRNA-Sec, transcribed by both Pol II and Pol III, was diminished to 78% of the control value. Pol II transcription of *α*-tubulin,* LmjF.06.0200*,* LmjF.06.0210*, and* LmjF.06.0370* was slightly affected. Interestingly, 18S rRNA signal was reproducibly reduced to ~55% of the control (Figures 5(a) and 5(b)). To further analyze this unexpected result, the 24S*β* rRNA gene was included in new nuclear run-on experiments. As shown in Figures 5(c) and 5(d), similarly to the 18S rRNA, transcription of the 24S*β* rRNA gene was reduced to 39% in the DKO+1 cell line. Transcription of the spliced-leader (SL) RNA was not affected (Figures 5(c) and 5(d)), supporting the previous results that indicate that LmBdp1 does not participate in Pol II transcription.

### 3.6. LmBdp1 Binds to Pol III Promoters

**Figure 6:**
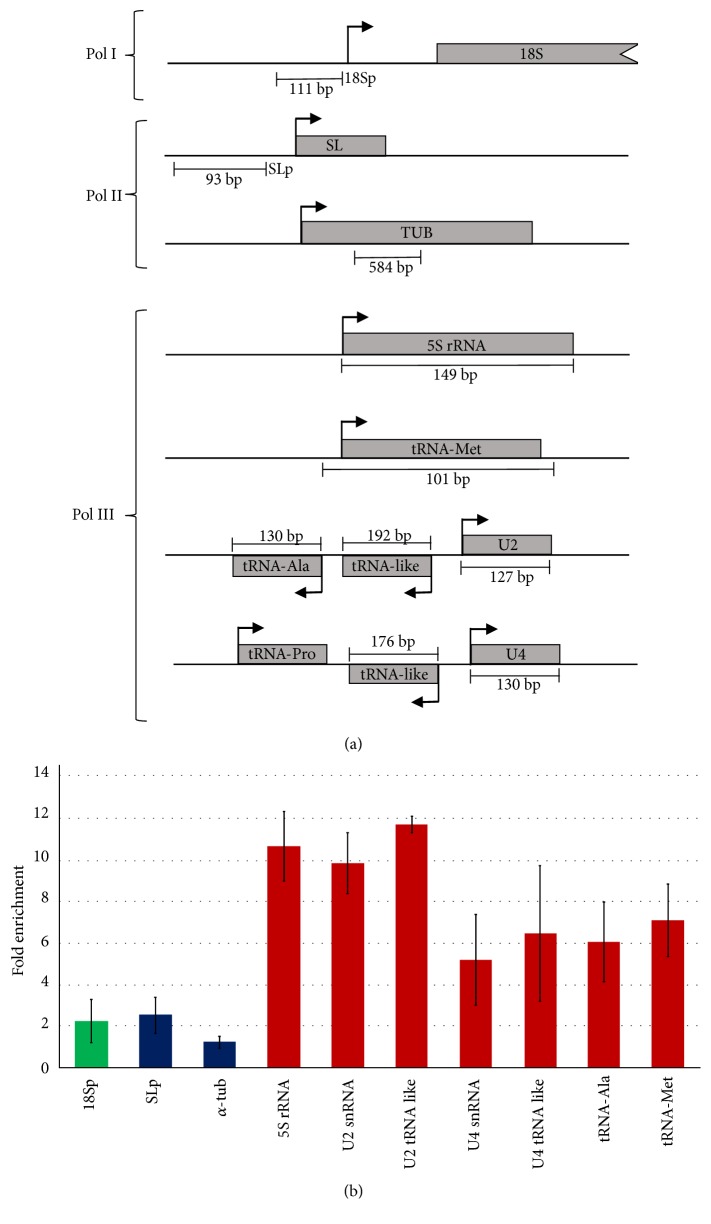
Chromatin immunoprecipitation analysis of LmBdp1. (a) Schematic representation of the genes and promoter regions examined by ChIP experiments. (b) Chromatin from a cell line that expresses the recombinant protein LmBdp1-PTP was precipitated with a ChIP-grade anti-Prot A antibody. Precipitated DNA was analyzed by qPCR. The results from three independent ChIP experiments, each analyzed by three qPCR reactions, are shown. Error bars indicate standard deviations. Results are presented as fold enrichment over negative control precipitations.

To determine whether LmBdp1 associates to Pol III promoter regions* in vivo*, chromatin immunoprecipitation (ChIP) experiments were carried out, using the* L. major* cell line that expresses LmBdp1 fused to the PTP tag. Immunoprecipitations were conducted with a ChIP-grade anti-Prot A antibody, which recognizes the two Prot A domains present in the PTP tag [41], and with a nonspecific mouse immune serum as negative control. To assess the binding of LmBdp1-PTP to the* L. major* genome, qPCR assays were performed with the purified DNA. Data obtained from three independent experiments showed that LmBdp1 is enriched in the 5S rRNA, tRNA-Ala, tRNA-Met, and the promoter regions from the U2 and U4 snRNAs (tRNA-like sequences) (Figure 6). Enrichment was also detected within the U2 snRNA gene, which contains an internal promoter element [25]. Thus, these results confirm the binding of LmBdp1 to Pol III promoters. As anticipated, enrichment was not observed with the *α*-tubulin gene and the SL RNA promoter region (Figure 6). Notably, we did not find association of LmBdp1 with the promoter region of the rRNA transcription unit, which suggests that the reduction in 18S and 24S*β* rRNA transcription that was observed in the LmBdp1 DKO+1 cell line (Figure 5) is an indirect effect.

## 4. Discussion

In this work, we characterized the orthologue of the Bdp1 subunit of transcription factor TFIIIB in* L. major*. The presence of Bdp1 in this ancient protozoan parasite indicates that the basic mechanisms of regulation of Pol III transcription were established early in the evolution of the eukaryotic lineages. Notably, the molecular mass of Bdp1 varies considerably across evolution. Whereas the predicted weights of isoforms 1 of Bdp1 in* Homo sapiens* and* Mus musculus* are 293.8 and 270.1 kDa, respectively, Bdp1 is predicted to be a ~34.1 kDa protein in* T. brucei* and a ~44 kDa protein in* L. major*. Molecular masses of 67.7 and 78.2 are predicted for Bdp1 in* Saccharomyces cerevisiae* and* Drosophila melanogaster *(isoform A), respectively. Sequence identity of LmBdp1 ranges from 19.8 to 22.8% for the yeast and human orthologues, respectively; higher identities were observed with Bdp1 from* T. brucei* (28.5%) and* T. cruzi* (35.3%) (Figure 1(a)). As expected, LmBdp1 is very similar to its orthologues in other species of* Leishmania*, showing identities that range from 79.7% (with* Leishmania panamensis*) to 95.9% (with* Leishmania gerbilli*) (Supplementary Figure 2).

In spite of molecular mass and sequence variations, Bdp1 orthologues share the extended SANT domain and other conserved sequences, including the N-terminal region, the long arm, and the tether region. LmBdp1 possesses the characteristic extended SANT domain, predicted to fold into five *α*-helices (Figure 1). Similarly to Bdp1 from yeast, a sixth *α*-helix that comprises the long arm is present in LmBdp1 (Supplementary Figure 1). Interestingly, the long arm is predicted to be present in all the Bdp1 orthologues that we analyzed, with the exception of the human protein (Supplementary Figure 1) [10]. In yeast, the long arm forms a coiled-coil structure with Brf1 homology domain II and ends next to winged helix domains 2 and 3 from Pol III subunit C34 [11]. The N-linker region is characterized by the presence of 3-6 aromatic amino acids (W, F, and Y) (Figure 1(a)). However, only the conserved W that is located at the end of the N-linker is present in LmBdp1 (Figure 1(a)). Since the missing aromatic residues anchor the N-linker to the major groove of the DNA through aromatic-sugar interactions with the DNA backbone [11], a different type of contacts should occur between the LmBdp1 N-linker and the promoter DNA. The Bdp1 tether, involved in interactions with some Pol III subunits, is folded into several *β*-sheet structures in* S. cerevisiae*,* H. sapiens*,* Schizosaccharomyces pombe*, and* D. melanogaster* (Supplementary Figure 1) [11, 12]. Nonetheless, in* L. major* and other trypanosomatid parasites this region is not predicted to fold into *β*-sheets (Supplementary Figure 1). Thus, LmBdp1 shares several characteristics with other Bdp1 orthologues, but it also shows some distinctive features.

Our data demonstrate that LmBdp1 participates in Pol III transcription, as the DKO+1 cell line showed reduced levels of tRNAs, 5S rRNA, and U2 snRNA in nuclear run-on experiments (Figure 5). The association of LmBdp1 with genes transcribed by Pol III was demonstrated by ChIP analysis (Figure 6). While nuclear run-on assays exhibited a reduction in the transcription levels of 18S and 24S*β* rRNAs, ChIP analysis did not reveal the binding of LmBdp1 to the promoter region of the ribosomal transcription unit. Thus, the observed decrease in 18S and 24S*β* rRNA transcription is most likely a secondary effect caused by the reduction of 5S rRNA, as cells coordinate the expression levels of all rRNA species to regulate ribosome biogenesis [42]. The target of rapamycin (TOR) signal-transduction pathway is a key regulator of this process [43]. Interestingly, while most eukaryotes possess one or two TOR kinases,* L. major* and other trypanosomatids have three different TOR kinases [44] that might coordinate transcription by all nuclear RNA polymerases.

We were unable to generate null mutants of LmBdp1, as the DKO+1 cell line contained an extra copy of LmBdp1 (Figure 3). It is possible that this additional copy was generated while trying to delete the second endogenous gene of LmBdp1, as attempts to knockout essential genes in* Leishmania* commonly produce alteration in gene copy number, either by gene amplification or changes in ploidy [33, 45–47]. Thus, this result strongly suggests that LmBdp1 is essential for the growth of* L. major* promastigotes, as has been reported in yeast [17]. Regardless of the moment when the extra LmBdp1 copy was produced, the growth of the DKO+1 cell line is strongly impaired and the expression of LmBdp1 is reduced by 70% (Figure 4). We tried to restore the expression levels of LmBdp1 in the DKO+1 cell line by transfecting it with the pLmBdp1-PTP vector (data not shown). However, several attempts to obtain transfected parasites were unsuccessful due to the inability of the DKO+1 cell line to reach the optimal cell densities for electroporation and to tolerate the drug selection process. As an alternative approach, we transfected the LmBdp1 single-knockout parasites with the pLmBdp1-PTP vector and then tried to knockout the second LmBdp1 allele (data not shown). Nevertheless, we were not capable of deleting the second endogenous copy of LmBdp1, which suggests that the expression of the recombinant LmBdp1-PTP protein in the resultant cell line was not high enough to allow the elimination of the second allelic copy of LmBdp1.

The function of Bdp1 is regulated by phosphorylation of specific amino acids. In logarithmically growing yeast cells, four Bdp1 residues (S49, S164, S178, and S586) are phosphorylated by PKA, Sch9, and CK2 kinases; and Bdp1 is dephosphorylated under conditions that reduce Pol III transcription [48]. Human Bdp1, by contrast, is phosphorylated by CK2 during mitosis to inhibit U6 snRNA transcription [49]. An* in silico* search allowed us to identify several potential phosphorylation sites in LmBdp1, including T308 (PhosTryp score of 0.933), S129 (score of 0.905), and S327 (score of 0.841) (Supplementary Figure 4). Notably, PKA [50] and CK2 [51] are present in* Leishmania*. Thus, similarly to yeast and human, the activity of LmBdp1 could be controlled by phosphorylation. This is supported by the fact that Western blot analysis of LmBdp1 often showed two or more bands (Figure 4(b) and Supplementary Figure 5) that might correspond to different phosphorylation states of the protein.

Besides its role in Pol III transcription initiation, Bdp1 is also involved in maturation of tRNAs by interacting with RNAse P, the enzyme required for the site-specific cleavage of the 5′ leader sequence of precursor tRNAs [17]. Also, Bdp1 participates in the integration of Ty1, a long terminal repeat retrotransposon, upstream of tRNA genes in* S. cerevisiae *[52]. Moreover, Bdp1 also interacts with Hmt1, a protein arginine methyltransferase from yeast that inhibits tRNA transcription by methylating an unknown factor of the Pol III complex [53]. Bdp1 seems to also participate in Pol II transcription termination of noncoding RNAs in the vicinity of tRNA genes [54]. It remains to be determined whether Bdp1 also performs multiple functions in* L. major* and other trypanosomatids.

## 5. Conclusions

In the present study, we show that LmBdp1 shares several sequence and structural features with Bdp1 orthologues from other species, such as the presence of the extended SANT domain and the long arm. But some differences were also observed, including the occurrence of only one aromatic residue in the N-linker and the lack of predicted *β*-sheet structures in the tether region of LmBdp1. Our data also demonstrate that LmBdp1 localizes to the nucleus, where it regulates the Pol III transcription of tRNAs, 5S rRNA, and U2 snRNA by associating with their promoter regions. Targeted gene replacement analysis strongly suggests that LmBdp1 is essential for the growth of promastigotes of* L. major*. Thus, LmBdp1 could be considered a suitable candidate for drug development against* Leishmania*.

## Data Availability

The data used to support the findings of this study are available from the corresponding author upon request.

## Supplementary Materials

Supplementary Materials Supplementary Figure 1: sequence and structure analyses of conserved regions of Bdp1. Supplementary Figure 2: sequence alignment of the N-linker and extended SANT domain in different species of* Leishmania*. Supplementary Figure 3: PCR analysis of LmBdp1 single- and double-knockout +1 parasites. Supplementary Figure 4: predicted phosphorylated residues in LmBdp1 using the PhosTryp web tool. Supplementary Figure 5: Western blot analysis of LmBdp1.
