# Supplementary material for: TFIIIB Subunit Bdp1 Participates in RNA Polymerase III Transcription in the Protozoan Parasite Leishmania major
Source: Biomed Res Int. 2019 Apr 1;2019:1425281. doi: 10.1155/2019/1425281 (PMC6463643; doi:10.1155/2019/1425281)
Supplement: Supplementary Materials — Supplementary Figure 1: sequence and structure analyses of conserved regions of Bdp1. Supplementary Figure 2: sequence alignment of the N-linker and extended SANT domain in different species of Leishmania. Supplementary Figure 3: PCR analysis of LmBdp1 single- and double-knockout +1 parasites. Supplementary Figure 4: predicted phosphorylated residues in LmBdp1 using the PhosTryp web tool. Supplementary Figure 5: Western blot analysis of LmBdp1. [file 1425281.f1.pdf]

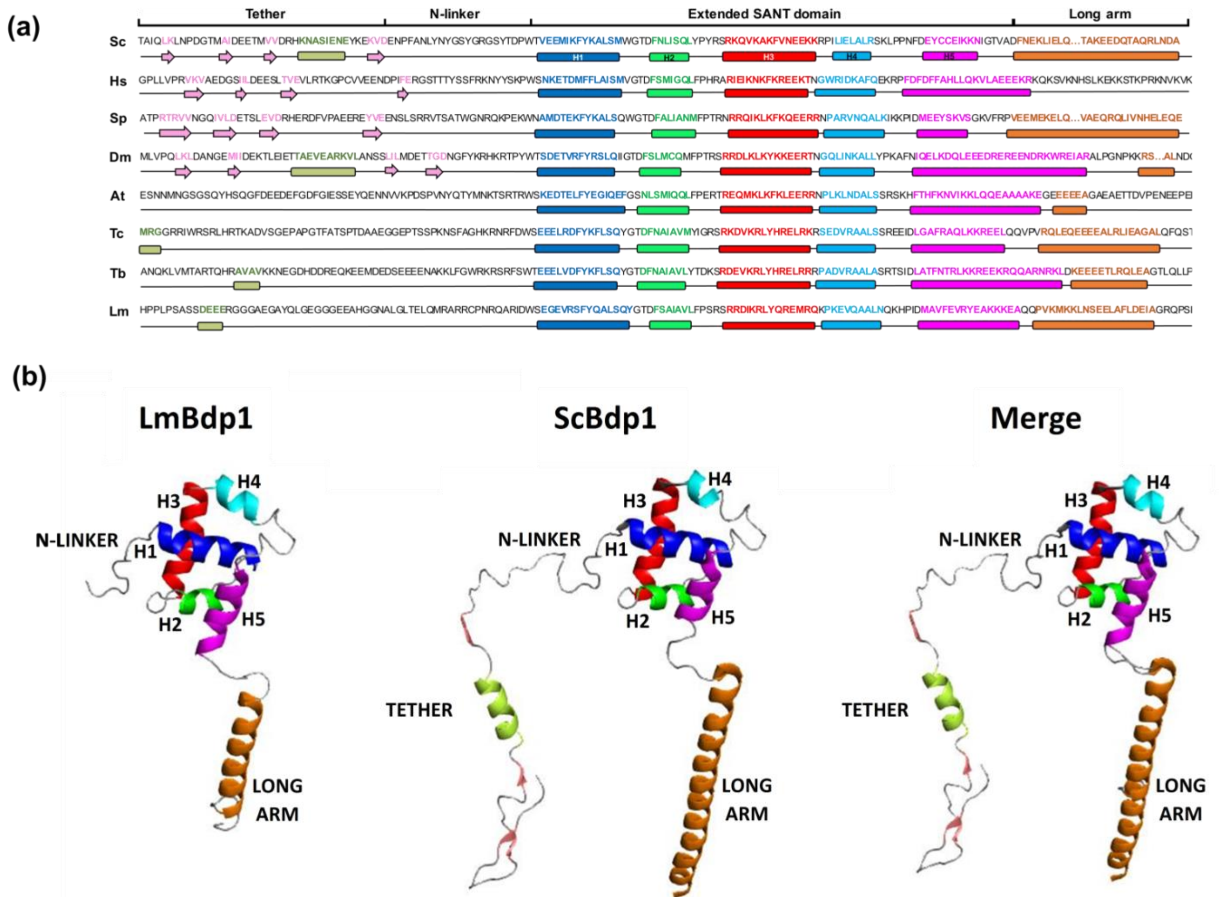

Suppl. Figure 1

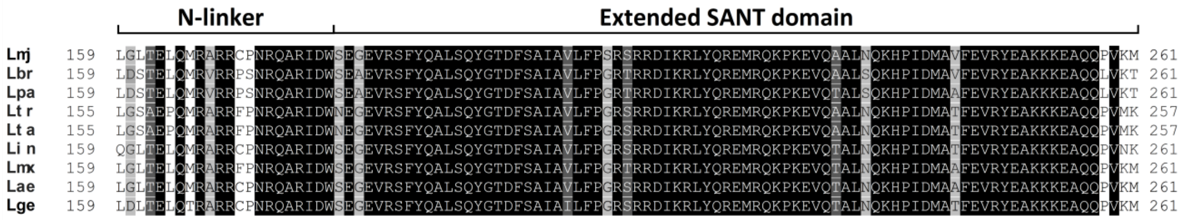

Suppl. Figure 2

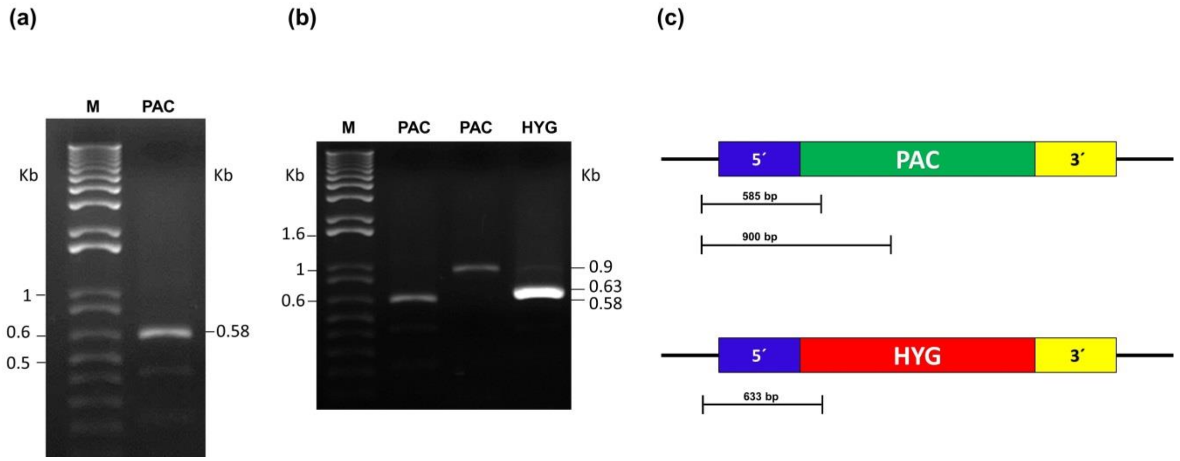

Suppl. Figure 3

(a)

Peptide	Pos	Score
GVEEATAAPPS	308	0.933
PLPSASSDEEE	129	0.905
THQADSEDGPE	327	0.841
LPSASSDEEER	130	0.830
HPPLPSASSDE	127	0.791
PRPSASVAPLP	97	0.765
AGRQPSESAMH	281	0.752
RLHQQSPTHSP	111	0.723
PVPAATLGGVE	300	0.651
KRPRGTHQADS	322	0.646

(b)

```
1   MDDNEFEFPPDQLDDVVANRLSPYQTVMPSPLRTPFAYPAANLPLSMLAQ 50
51  IPLEHLPPTQPKNPQNFSIGQVLRGALSTSHMRAPPGGSRMPRPSASVAP 100
101 LPHSQRLHQQSPTHSPYLSAGHPPLPSASSDEEERGGGAEGAYQLGEGGG 150
151 EEAHGGNALGLTELQMRARRCPNRQARIDWSEGEVRSFYQALSQYGTDFS 200
201 AIAVLFPSRSRRDIKRLYQREMRQKPKEVQAALNQKHPIDMAVFEVRYEA 250
251 KKKEAQQPVKMKKLNSEELAFLDEIAGRQPSESAMHMKGEELEVPVPAAT 300
301 LGGVEEATAAPPSRLRKRPRGTHQADSEDGPESKRATKEIPPAADDDFDM 350
351 EQESFFDMVVRHEREDNAPLDMLFAAQLEQGQQQHLAALEDSDFSFE    397
```

Tether
N-linker
Extended SANT
Long arm

Suppl. Figure 4

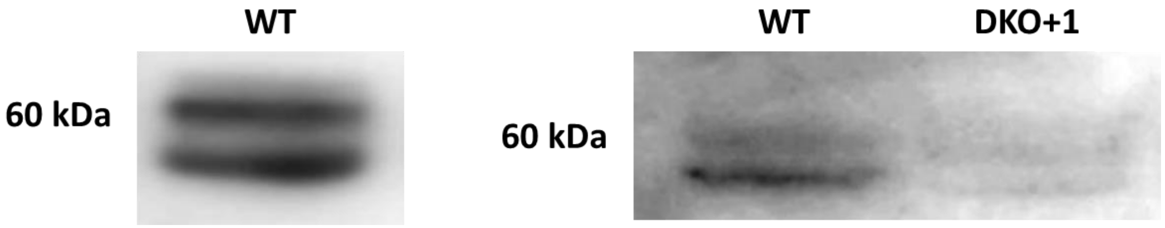

Suppl. Figure 5

Supplementary Figure 1. Sequence and structure analyses of conserved regions of Bdp1. **(a)** Sequence and predicted secondary structure analysis of the Tether, N-linker, extended SANT and Long arm domains of Bdp1 from *S. cerevisiae* (Sc), *H. sapiens* (Hs), *S. pombe* (Sp), *D. melanogaster* (Dm), *A. thaliana* (At), *T. cruzi* (Tc), *T. brucei* (Tb) and *L. major* (Lm). Predicted secondary structure elements are shown below each sequence. The β-strands are denoted by pink arrows, whereas α-helices by colored rectangles. The five α-helices of the extended SANT domain are labeled as H1 to H5. **(b)** Predicted three-dimensional structure of the extended SANT domain and flanking regions of LmBdp1 by homology-modeling using the crystal structure of *S. cerevisiae* Bdp1 (ScBdp1) as a template. The colors of the structural elements are conserved in panels (a) and (b).

Supplementary Figure 2. Sequence alignment of the N-linker and extended SANT domain in different species of *Leishmania*. Identical residues in all species are indicated by black shading. Conserved substitutions are denoted by dark-grey shading with white lettering and semi-conserved substitutions are indicated by light-grey shading with black lettering, according to the Clustal Ω program. The species analyzed are: *L. major* (Lmj), *L. braziliensis* (Lbr), *L. panamensis* (Lpa), *L. tropica* (Ltr), *L. tarentolae* (Lta), *L. infantum* (Lin), *L. mexicana* (Lmx), *L. aethiopica* (Lae), *L. gerbilli* (Lge).

Supplementary Figure 3. PCR analysis of LmBdp1 single- and double-knockout +1 parasites. **(a)** Using genomic DNA from the single-knockout cell line as template, a 585-bp fragment was amplified from the mutant locus where a copy of LmBdp1 was substituted with the *pac* gene**. (b)** With genomic DNA from the double-knockout +1 cell line as template, fragments of 585 and 900 bp were amplified from the mutant locus where a copy of LmBdp1 was replaced with the *pac* gene; and a 633 bp fragment was amplified from the

mutant locus where a copy of LmBdp1 was substituted with the *hyg* gene. The size marker corresponds to a 1-kb ladder (Invitrogen) (lane M). **(c)** Map of the mutant loci showing the location of the PCR products presented in panels (a) and (b). The expected size of the fragments is also indicated.

Supplementary Figure 4. Predicted phosphorylated residues in LmBdp1 using the PhosTryp web tool. The highest-scoring serine and threonine residues are indicated (a). LmBdp amino acid sequence showing the predicted phosphorylated sites in red and underlined. The location of the conserved domains is also indicated (b).

Supplementary Figure 5. Western blot analysis of LmBdp1. Wild-type (WT) and double-knockout +1 (DKO+1) *L. major* parasites were analyzed using the LmBdp1 antibody.
